# The location of “8”-shaped hatching influences inner cell mass formation in mouse blastocysts

**DOI:** 10.1371/journal.pone.0175150

**Published:** 2017-04-06

**Authors:** Yohei Onodera, Kazumasa Takahashi, Mayumi Goto, Mibuki Anzai, Natsuki Ono, Hiromitsu Shirasawa, Wataru Sato, Hiroshi Miura, Naoki Sato, Akira Sato, Yukiyo Kumazawa, Yukihiro Terada

**Affiliations:** Department of Obstetrics and Gynecology, Akita University Graduate School of Medicine, Akita, Japan; Rutgers University Newark, UNITED STATES

## Abstract

The hatching of a blastocyst where the blastocyst portions on the inside and the outside of the zona pellucida feature a figure-of-eight shape is termed “8”-shaped hatching; this type of hatching has been reported to affect the proper presentation of the inner cell mass (ICM) in both human and mouse embryos. Here, our aim was to investigate the factors that affect ICM presentation during “8”-shaped hatching. We performed IVF by using B6D2F1 female mice and ICR male mice, and used the 104 captured blastocysts. Embryos were maintained in KSOM at 37°C in a 5% CO_2_, 5% O_2_, and 90% N_2_ environment, and their growth behavior was monitored individually and continuously using time-lapse cinematography. At 120 h after insemination, embryos were immunostained and examined under a confocal microscope. We used the hatching form to identify “8”-shaped hatching, and we classified the “8”-shaped-hatching blastocysts into two groups, one in which the hatching site was near the ICM center, and the other in which the hatching site was far from the ICM center. We measured each group for ICM size and the number of Oct3/4-positive cells. Of the 95 hatching or hatched embryos, 74 were “8”-shaped-hatching blastocysts, and in these embryos, the ICM was significantly wider when the hatching site was near the ICM than when the hatching site was far from the ICM (*P* = 0.0091). Moreover, in the “8”-shaped-hatching blastocysts in which the ICM was included in the blastocyst portion outside the zona pellucida―the portion defined as the “outside blastocyst”―after the collapse of this outside blastocyst, the ICM adhered to the trophectoderm of the outside blastocyst, opposite the hatching site. Our results indicate that in “8”-shaped-hatching blastocysts, the hatching site and the collapse of outside blastocyst affect ICM formation. Thus, the assessment of “8”-shaped hatching behaviors could yield indices for accurately evaluating embryo quality.

## Introduction

The selection of healthy, high-quality embryos plays a crucial role in assisted reproductive technology (ART) success. One of the qualitative evaluations of embryos is morphological assessment, and in the case of human embryos, the Gardner criteria are widely used for this purpose [1]. A change in the morphology of the inner cell mass (ICM) exerts various effects on embryo prognosis. For example, the scattered ICM induced following treatment with an inhibitor of Rho-associated kinase activity was shown to increase fetal loss in mouse [2]. Attention has also been focused on the hatching mode for its effect on ICM morphology. Hatching modes have thus far been classified based on the site of hatching or on factors such as the zona pellucida (ZP) slit mode [3]. The ZP internal and external areas (i.e., the blastocyst portions inside and outside the ZP) adopt a hatching morphology similar to the figure “eight”; this “8”-shaped hatching has attracted attention as one factor for the qualitative evaluation of embryos, and for its effect in elongating the hatching period [4]. Moreover, in the case of mouse embryos, “8”-shaped hatching has been suggested to affect ICM presentation [5]. Furthermore, the relationship between the hatching site and hatching progress has also been investigated [6].

The development of time-lapse monitoring (TLM) technology has enabled less invasive and more detailed embryo observation than previously possible. TLM has not only allowed real-time examination of the embryo condition: it has also enabled dynamic analysis of embryos based on continuous observation. One of the factors used for TLM-based embryo evaluation is blastocyst collapse, although the clinical significance of this remains debated [7, 8]. However, a relationship between blastocyst collapse and monozygotic twinning (MZT) has been reported [9], with blastocyst collapse being considered to be a behavior that affects ICM morphology.

The change in ICM morphology that occurs in “8”-shaped hatching has long been suspected to be due to herniation where the ICM passes through the ZP hole [10], but the underlying details remain to be clarified. Here, we aimed to elucidate the factors that induce changes in ICM morphology in “8”-shaped hatching. In embryos that were identified as those undergoing “8”-shaped hatching, we focused on two factors, hatching site and blastocyst collapse, and investigated their effect on ICM size. Moreover, in the case of blastocyst collapse, which is already recognized to occur inside the ZP, we examined the collapse of the blastocyst portion outside the ZP, a unique behavior observed in “8”-shaped hatching, and investigated its effects on ICM morphology.

## Material and methods

### Collection of mouse oocytes, zygotes, and embryos

All animal experiments were performed in accordance with the Guide for Care and Use of Laboratory Animals of Akita University. All mice were housed in cages at 23°C with a photoperiod of 12 h light and 12 h darkness with free access to food and water. In the ICM evaluation performed here, the developmental stage had to be established as precisely as possible. Therefore, instead of the tubal perfusion method, IVF was selected as an embryo-acquisition method: Female B6D2F1 mice aged 12–13 weeks old were intraperitoneally injected with 5 IU of serum gonadotrophin (Serotropin; ASKA Animal Health Co, Tokyo, Japan) and, after 48 h, with 5 IU of human chorionic gonadotrophin (Mochida Pharmaceutical Co, Tokyo, Japan) to induce ovulation. After a further 14 h, female mice were euthanized by cervical dislocation, which was performed by a highly skilled technician, and oocytes were collected from these unmated mice and fertilized, using IVF, with ICR mouse sperm in human tubal fluid (Irvine Scientific, Santa Ana, CA, USA) medium containing 0.1% bovine serum albumin (BSA; Wako Pure Chemical, Osaka, Japan). Male mice were euthanized by cervical dislocation 2 h before insemination, and their spermatozoa were retrieved from the cauda epididymis by squeezing. The retrieved spermatozoa were then suspended in HTF medium supplemented with 0.5% BSA and incubated at 37°C in 5% CO_2_ and 95% air for 2 h and transferred to the IVF medium (at a final concentration of 200 sperm/μl).

### Embryo culture

At 4 h after insemination, IVF embryos were transferred into KSOM (Ark Resource, Kumamoto, Japan) and cultured in a 150-μl drop of medium on a Primo Vision Embryo Culture Dish (Vitrolife, Gothenburg, Sweden), which was then covered with mineral oil. Embryos were cultured under these conditions: 5% O_2_, 5% CO_2_, 90% N_2_, and 37.0°C. Culture media were not replaced during the experiments.

### Time-lapse imaging and analysis of blastocyst hatching

**Fig 1:**
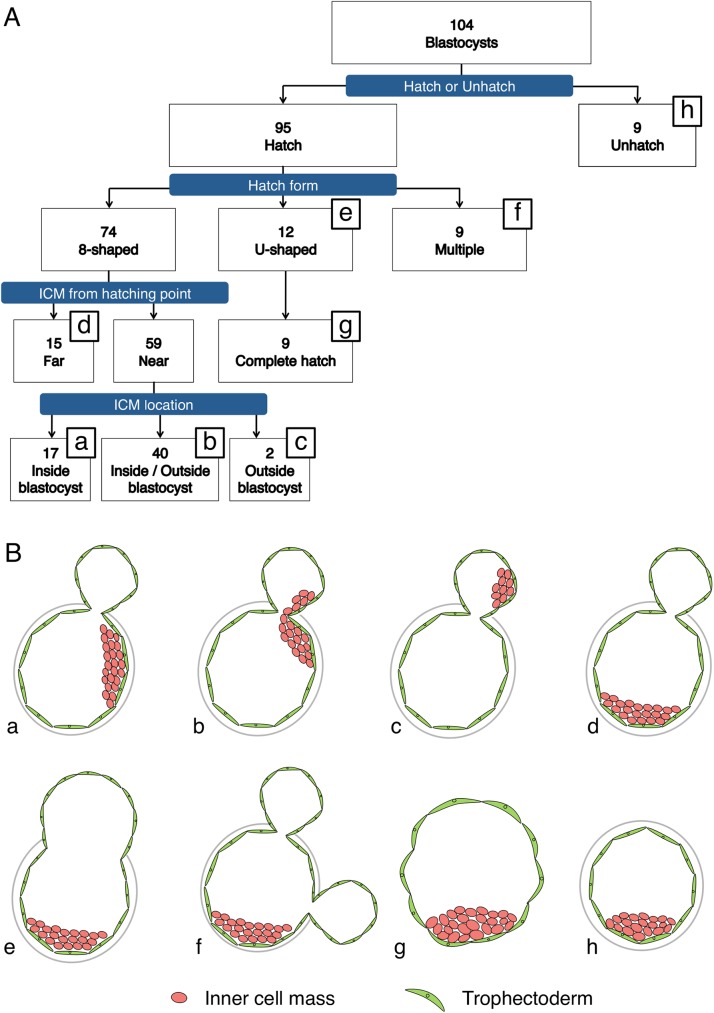
Sorting and evaluation of blastocysts. Flowchart (**A**) and schematic of the morphology at 120 h post-insemination (**B**) of the 104 blastocysts obtained in IVF. Based on morphology, the embryos can be classified into 8 types (**B**, a-h). Hatching embryos, based on their morphology, can be classified into “8”-shaped-hatching (a-d), U-shaped-hatching (e), and multiple-hatching-site (f) blastocysts. Only 9 of the U-shaped-hatching blastocysts hatched completely (g). The “8”-shaped-hatching blastocysts were classified according to the relationship between the hatching site and inner cell mass (ICM) position into the Near group (a-c) and Far group (d). The Near-group embryos were further classified into embryos in which the ICM was located both inside the zona pellucida (ZP) and in the outside blastocyst (b); embryos in which all of the ICM was included inside the ZP (a); and embryos in which all of the ICM was included inside the outside blastocyst (c). The term “outside blastocyst” refers exclusively to the specific portion of the blastocyst that lies outside the ZP.

Embryos were imaged using a TLM system (Primo Vision, Vitrolife) at 5-min intervals, and were examined until 120 h post-insemination based on referring to previous work [5]. The embryos were continuously evaluated and the collected TLM data were used for sorting the embryos according to the hatching mode (Fig 1A); as per the criteria reported in previous work [5], the hatching modes were differentiated as follows: When blastocysts featured 2 blastocoels divided by the ZP and the ZP-opening size was ≤25 μm, they were regarded as “8”-shaped-hatching blastocysts (Fig 1B(a-d)). Conversely, when the opening size increased to >25 μm during the monitoring period, or when the newly generated openings of the blastocysts were >25 μm in diameter, the blastocysts were regarded as U-shaped-hatching blastocysts (Fig 1B(e)). Embryos harboring multiple small-diameter hatching points (Fig 1B(f)) were excluded from this study.

### Definition and measurement of blastocyst collapse

In “8”-shaped hatching, the portions of the blastocyst that lie inside and outside the ZP have been termed “inside blastocyst” and “outside blastocyst,” respectively. Moreover, a collapse occurring in the area inside the ZP is called an “inside collapse,” and a collapse occurring in the area outside the ZP (i.e., a collapse of the “outside blastocyst”) is called an “outside collapse.”

**Fig 2:**
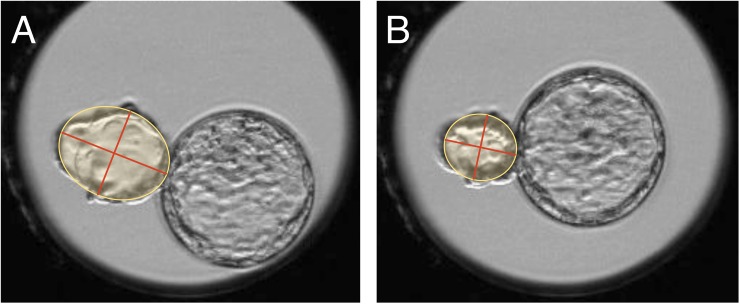
Collapse detection method. The outside blastocyst immediately before (**A**) and immediately after (**B**) collapse was set in an ellipse and the lengths of the long and short axes were measured in order to calculate the cross-sectional area. An area reduction of ≥50% was defined as a collapse.

In previous work, a blastocyst was considered to have undergone a collapse if a comparison of the ZP lumen before and after the collapse showed that its cross-section had decreased by ≥50% [7], and this method was used for assessing inside collapse. For outside collapse, ZP cannot be used as an index marker, and thus we compared the outside blastocyst area before and after collapse. To determine the area, we used the “Primo Vision analyzer” drawing tool. We set the outside blastocyst cross-section as an ellipse, and then obtained the area before and after collapse; thus, area = long axis/2 × short axis/2 × π (Fig 2). We calculated the ratio (%) of the area before and after blastocyst collapse, and defined a collapse as an area reduction of ≥50%. In this manner, we investigated whether outside and inside collapse occurred or not.

### Antibodies

Embryos were stained with the following antibodies: mouse monoclonal anti-Cdx2 (CDX2-88; BioGenex, Fremont, CA, USA), goat polyclonal anti-Oct3/4 (Santa Cruz Biotechnology, San Francisco, CA, USA), mouse monoclonal anti-Na^+^/K^+^-ATPase α-1 (C464.6; Upstate Biotechnology, Lake Placid, NY, USA), and rabbit polyclonal anti-ZO2 (Invitrogen, Carlsbad, CA, USA).

### Immunofluorescence and confocal microscopy

At 120 h after insemination, embryos were immunostained.

For Oct3/4 and Cdx2 staining, embryos were fixed in 3.7% formaldehyde for 30 min at room temperature and then washed thrice for 45 min in wash buffer-1 (D-PBS containing 0.1% BSA; Sigma-Aldrich, St. Louis, MO, USA). Next, the embryos were permeabilized by placing them in wash buffer-2 (D-PBS containing 0.5% Triton X-100 (Sigma-Aldrich) and 0.1% BSA) for 30 min at room temperature. After washing thrice in buffer-1, the embryos were blocked with D-PBS containing 3% BSA for 1 h at room temperature. After blocking, embryos were incubated with anti-Oct3/4 (1:50) primary antibodies overnight at 4°C, washed thrice in wash buffer-2, and then incubated with secondary antibodies (1:200; Alexa Flour 647-conjugated donkey anti-goat, Abcam) for 30 min at room temperature. After washing thrice in buffer-2, embryos were incubated with anti-Cdx2 (ready-to-use) for 1 h at room temperature, washed thrice in buffer-2, and then incubated with secondary antibodies (1:200; Alexa Flour 488-conjugated goat anti-mouse IgG, Invitrogen) for 30 min at room temperature. Following another 3 washes, chromatin was stained with 1:1000 Hoechst 33342 (Dojindo Molecular Technologies, Kumamoto, Japan) for 20 min at room temperature.

For ZO2 and Na^+^/K^+^-ATPase staining, embryos were fixed in cold methanol for 15 min and then permeabilized and blocked using PHEM buffer (60 mM Pipes, 25 mM Hepes, 10 mM EGTA, 1 mM MgCl_2_, pH 6.9) containing 0.01% Triton X-100 and 3% BSA for 45 min at room temperature. After blocking, embryos were incubated with anti-ZO2 (1:200) primary antibodies overnight at 4°C, washed thrice in wash buffer-2, and then incubated with secondary antibodies (1:200; Alexa Flour 647-conjugated goat anti-rabbit IgG, Invitrogen) for 30 min at room temperature. After washing thrice in buffer-2, embryos were incubated with anti-Na^+^/K^+^-ATPase α-1 (1:200) for 1 h at room temperature, washed thrice in buffer-2, and then incubated with secondary antibodies (1:200; Alexa Flour 488-conjugated goat anti-mouse IgG, Invitrogen) for 30 min at room temperature. Following another 3 washes, chromatin was stained with 1:1000 Hoechst 33342 (Dojindo Molecular Technologies, Kumamoto, Japan) for 20 min at room temperature.

**Fig 3:**
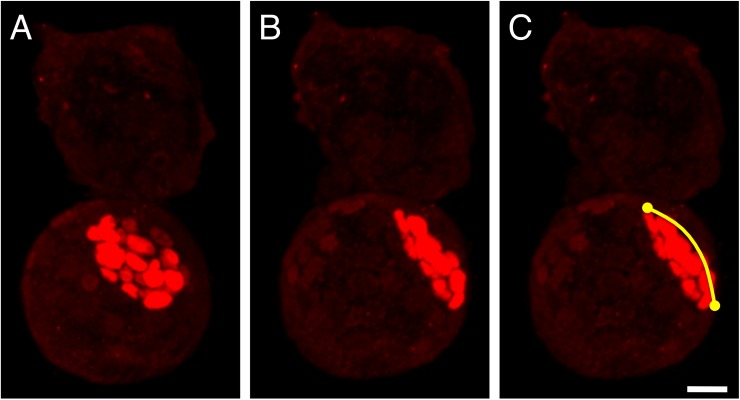
Method used for determining ICM size. Oct3/4 (red) was stained with specific antibodies, and Oct3/4-positive cell masses were identified as ICM. The 3D confocal image of each blastocyst was rotated to set the ICM center on the cross-section (**A, B**), and then the ICM end-to-end distance along the curved surface (**C**) was measured by following each of the perpendicular lines (curves) on the blastocyst image. Subsequently, these measurements for each blastocyst were averaged to obtain the “width” as an indicator of the size of the ICM in the blastocyst. Scale bar, 20 μm.

Fully processed embryos were mounted in glass-bottom dishes (Matsunami Glass Ind., Ltd, Osaka, Japan). Stained embryos were examined and measured using an LSM780 confocal laser-scanning microscope (Carl Zeiss AG, Jena, Thuringia, Germany). For obtaining confocal images, we used a 40× lens and acquired 1-μm-thick optical sections. To measure the ICM width (as an indicator of ICM size; described below), we used ZEN software (Carl Zeiss AG); Oct3/4-positive cell masses were identified as ICMs and Cdx2-positive cells were identified as the trophectoderm (TE). We used 3D images to measure the ICM width and count the Oct3/4-positive cells. ICM width (size) was assessed as shown in Fig 3: Images of the blastocysts were first rotated in order to set the ICM center at the middle of the cross-section, and then for each blastocyst, the end-to-end distance across the ICM was measured along the curved surface by following each of the two perpendicular lines indicated on the blastocyst image (these lines are curves when the blastocysts are rotated onto their side). After measuring the end-to-end distance along these two lines, the average was calculated to obtain the “ICM width” (the size measurement), which was then used for statistical analysis.

### Classification of “8”-shaped-hatching blastocysts by hatching location

**Fig 4:**
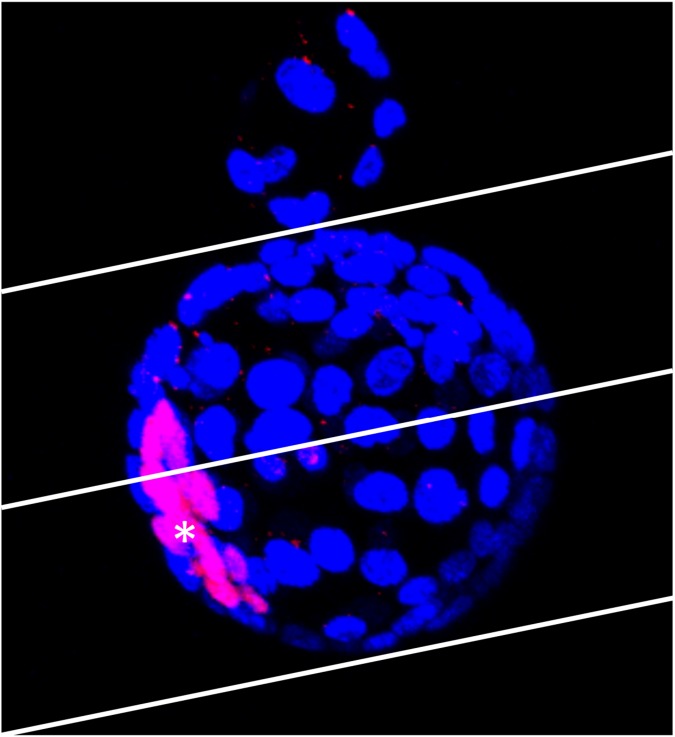
Classification of Near and Far groups. The ICM center (*) was identified and blastocysts were divided into two halves as demarcated in the figure, with the hatching site set as a vertex. Embryos with the hatching sites on the nearer side and the farther side from the ICM center were sorted into the “Near group” and “Far group,” respectively. The embryo shown in this figure was sorted into the Far group.

We classified the “8”-shaped-hatching blastocysts according to hatching site and based on their positional relationship with the ICM. As shown in Fig 4, the inside blastocyst was demarcated into two halves, with the hatching area serving as a vertex, and the embryos in which the hatching site was on the nearer side of the ICM center were defined as the “Near group” (Fig 1B(a-c)) and the embryos in which the hatching site was on the farther side were defined as the “Far group” (Fig 1B(d)). The Near group was further classified into embryos in which the ICM was both in the outside and inside blastocyst (Fig 1B(b)), only included in the inside blastocyst (Fig 1B(a)), or only included in the outside blastocyst (Fig 1B(c)). The 42 embryos in which the ICM was included in the outside blastocyst (Fig 1B(b and c)) were excluded from the ICM size measurement because of the difficulty in their classification.

### Statistical analysis

We used SPSS version 22.0 (SPSS Inc., Chicago, IL, USA) to analyze the data. We used Fisher’s exact test to examine the collapse-occurrence rate according to hatching morphology, and the Mann–Whitney U test to study the number of collapses. Student’s *t* test or Welch’s *t* test was used to study the effect of hatching morphology, hatching position, and outside collapse on ICM morphology and cell count. The correlation between the number of outside collapses and ICM size was studied using Spearman’s rank correlation test. *P*<0.05 was considered statistically significant. Data are presented as means ± SEM unless indicated otherwise.

### Ethical approval

The study was approved by the Institutional Animal Care and Use Committee of Akita University (Permission number: 28-2-4) and conducted according to the Akita University Animal Experimentation Regulations.

## Results

### Embryo growth and mode of hatching

**Fig 5:**
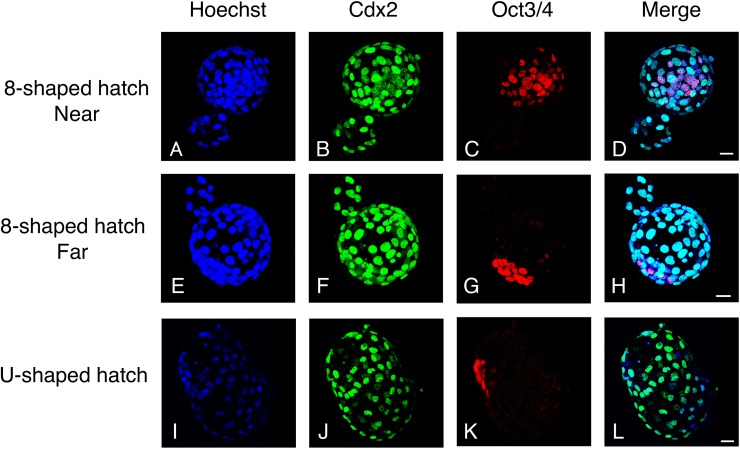
Immunostaining of “8”-shaped-hatching and U-shaped-hatching blastocysts. Cdx2 (green) and Oct3/4 (red) were stained with specific antibodies, and nuclei were stained with Hoechst (blue). In the case of “8”-shaped hatching, the figure shows embryos in the Near group (**A–D**) and Far group (**E–H**). Whereas the ICM tended to be morphologically compact in the Far group (**G**), it was scattered in the Near group (**C**; see Fig 7C).

**Fig 6:**
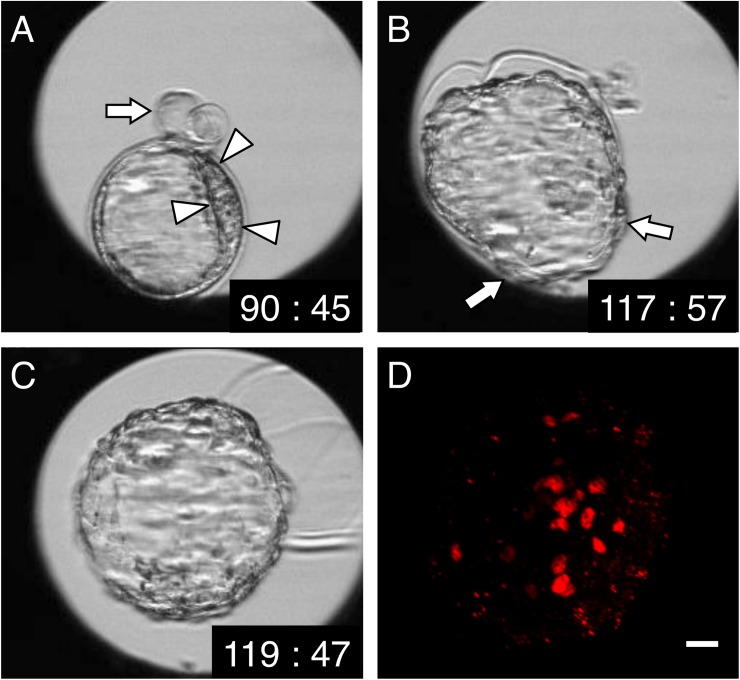
Time-lapse and immunostaining images of a U-shaped-hatching blastocyst that hatched completely, in which an initial “8”-shaped hatching occurred near the ICM. (**A**) At 90 h 45 min after insemination. Near the ICM (surrounded by arrowheads), an “8”-shaped hatch appears (arrow). (**B**) At 117 h 57 min after insemination. U-shaped hatching appears (arrow) in this embryo. (**C**) At 119 h 47 min after insemination. The embryo has hatched completely. (**D**) Immunostaining at 120 h post-insemination. The result confirmed that the ICM, stained by anti-Oct3/4 (red), is scattered. Scale bar, 20 μm.

Of the 156 embryos retrieved, 120 had attained the blastocyst stage at Day 5, and 104 of the 120 embryos were immunostained and their hatching morphology was evaluated using TLM and immunohistochemistry. Among the 95 hatching blastocysts, “8”-shaped-hatching blastocysts (Fig 1B(a-d), S1 Movie) accounted for 77.9% (74/95) and U-shaped-hatching blastocysts (Fig 1B(e), S2 Movie) for 12.6% (12/95). When we classified the 74 “8”-shaped-hatching blastocysts into the Near group (Figs 1B(a-c) and 5A–5D) and the Far group (Figs 1B(d) and 5E–5H), we found that the Near group accounted for 79.7% (59/74), and that the ICM was included in the outside blastocyst of 71.2% (42/59) of the Near-group embryos (Fig 1B(b and c)). Furthermore, in the TLM performed here, in certain “8”-shaped-hatching blastocysts in which the hatching started near the ICM, the embryos were observed to pass through U-shaped hatching before blastocysts hatched completely (Fig 6).

### Outside collapse occurs frequently in “8”-shaped-hatching blastocysts

Evaluation of the behavior of “8”-shaped-hatching blastocysts by using TLM confirmed that collapse was also induced in the outside blastocyst (S1 Movie). Thus, we next assessed the occurrence of collapse in “8”-shaped-hatching, U-shaped-hatching, and unhatched blastocysts (Table 1): The rate of inside collapse was significantly higher in unhatched blastocysts than in other blastocysts. In the case of “8”-shaped-hatching blastocysts, outside collapse was recorded in nearly 85% of the blastocysts but no inside collapse was observed. A comparison of the numbers of inside collapse in unhatched blastocysts and outside collapse in “8”-shaped-hatching blastocysts, 0 (0–1) and 2 (1–4) (median (IQR)), respectively, revealed that the number of outside collapse in “8”-shaped-hatching blastocysts was significantly higher.

**Table 1:** Rate and median of inside/outside collapse in “8”-shaped-hatching, U-shaped-hatching, and unhatched blastocysts.

		“8”-shaped hatching (*n* = 74)	U-shaped hatching (*n* = 12)	Unhatched (*n* = 9)
**Inside collapse**	**Rate**	0.0% (0/74)^a^	8.3% (1/12)^a^	44.4% (4/9)^a^
	**Median (IQR)**	0 (0–0)	0 (0–0)	0 (0–1)^b^
**Outside collapse**	**Rate**	86.5% (64/74)	-	-
	**Median (IQR)**	2 (1–4)^b^	-	-

^a^ The rate of inside collapse was higher in unhatched blastocysts than in other blastocysts (*P* = 0.0020, Fisher’s exact test).

^b^ Outside collapse in “8”-shaped-hatching blastocysts occurred significantly more frequently than inside collapse in unhatched blastocysts (*P* = 0.0007, Mann–Whitney U test).

### Factors influencing ICM structure

**Fig 7:**
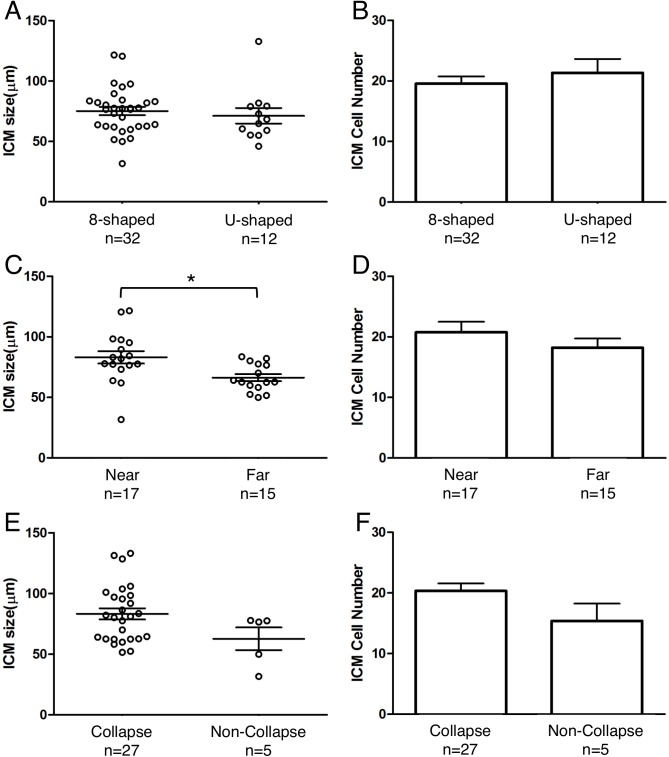
ICM size and cell number at 120 h post-insemination, sorted according to type of developmental behavior of blastocysts: “8”-shaped hatching and U-shaped hatching, hatching position relative to the ICM, and occurrence of outside collapse. (**A**, **B**) Comparison by hatching mode. (**C**, **D**) Comparison by hatching position. The ICM was significantly wider in the Near group than in the Far group (*P = 0.0091, Welch’s *t* test). (**E**, **F**) Comparison based on occurrence of outside collapse.

**Fig 8:**
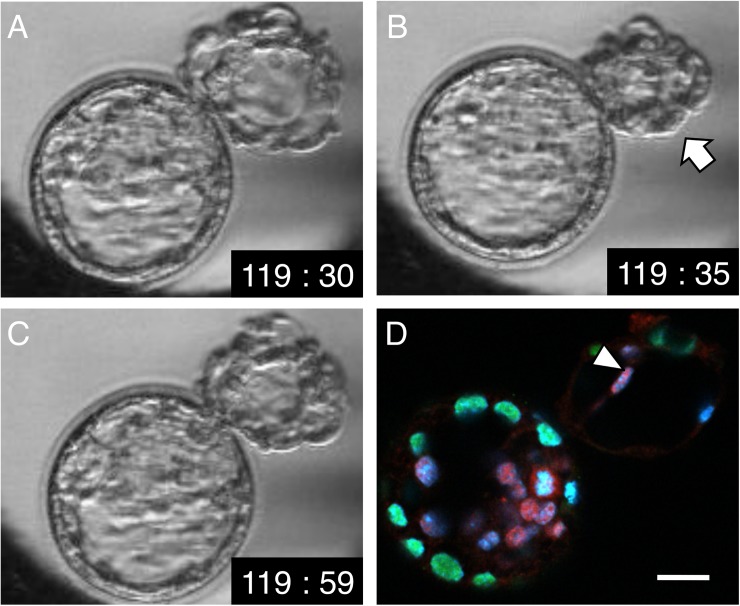
Time-lapse and immunostaining images of “8”-shaped hatching. (**A**) At 119 h 30 min post-insemination. The outside blastocyst is expanded. (**B**) At 119 h 35 min post-insemination. The outside blastocyst has collapsed (arrow). (**C**) At 119 h 59 min post-insemination. The outside blastocyst has re-expanded. (**D**) Immunostaining at 120 h post-insemination, immediately after the time point shown in **C**. A merged image of Oct3/4 (red), Cdx2 (green), and Hoechst (blue) staining is shown. Oct3/4-positive cells (arrowhead) were attached to the TE of the outside blastocyst opposite the hatching site. Scale bar, 20 μm.

The “8”-shaped-hatching blastocysts and U-shaped-hatching blastocysts showed no significant differences in ICM size (width: 75.2 ± 3.4 μm versus 71.2 ± 6.4 μm, *P* = 0.5617; Fig 7A) and the number of Oct3/4-positive cells (20 ± 1 versus 21 ± 2, *P* = 0.4573; Fig 7B). In our examination of the effect of hatching position, we found that the ICM was significantly wider in the Near group than in the Far group (83.1 ± 5.2 μm versus 66.3 ± 2.9 μm, *P* = 0.0091; Fig 7C). However, the Near and Far groups showed no significant differences in the number of Oct3/4-positive cells (21 ± 2 versus 18 ± 2, *P* = 0.2782; Fig 7D). Furthermore, after sorting based on the rate of occurrence of outside collapse, no significant difference was observed in ICM width (77.5 ± 3.5 μm versus 62.7 ± 9.4 μm, *P* = 0.1124; Fig 7E) or cell number (20 ± 1 versus 15 ± 3, *P* = 0.1254; Fig 7F), and the outside-collapse count was not significantly correlated with ICM size (*P* = 0.4639). Lastly, in two blastocysts among embryos that included the ICM in the outside blastocyst (as depicted in Fig 1B(b)), in the blastocysts that exhibited outside collapse (Fig 8), immunostaining revealed that Oct3/4-positive cells adhered to the TE of the outside blastocyst, opposite the hatching site (Fig 8D, S3 Movie).

### Proteins associated with the TE structure of “8”-shaped-hatching blastocysts

**Fig 9:**
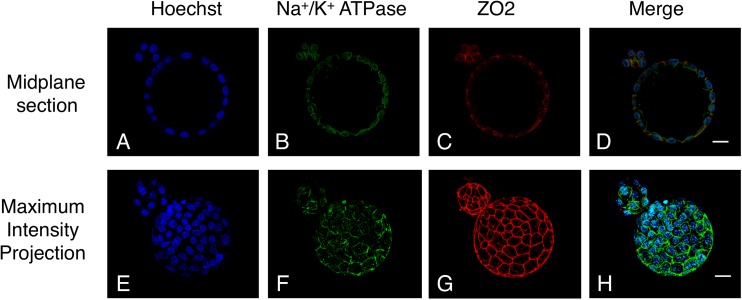
Immunostaining of an “8”-shaped-hatching blastocyst at 120 h post-insemination. The images show ZO2 (red) and Na^+^/K^+^-ATPase (green) staining, nuclei stained with Hoechst (blue), and the midplane section (**A–D**) and maximum intensity projection (**E–H**). The two TE-structure proteins Na^+^/K^+^-ATPase and ZO2 were similarly expressed inside and outside the ZP. Scale bar, 20 μm.

The “8”-shaped-hatching blastocysts were double-stained for Na^+^/K^+^-ATPase (Fig 9B & 9F) and ZO2 (Fig 9C & 9G). These two TE-structure proteins were expressed in both inside and outside blastocysts. In maximum intensity projections, we confirmed the same belt-like structure of ZO2 in both inside and outside blastocysts (Fig 9G).

## Discussion

In this study, we examined the relationship between “8”-shaped hatching and ICM morphology. We focused on the hatching location and blastocyst collapse, and evaluated them by using TLM and immunostaining. We report that, one, the ICM was significantly wider when the hatching site was near the ICM than when it was far from the ICM; and two, in “8”-shaped-hatching blastocysts containing ICM in the outside blastocysts, an outside collapse triggered separation of the ICM. These findings revealed that the hatching site and outside collapse affected ICM morphology.

We suspect that in the “8”-shaped-hatching blastocysts in which the hatching site was near the ICM, the blastocyst attempted to escape to the ZP outside through “8”-shaped hatching, and, consequently, the ICM was stretched and spread. In this study, after “8”-shaped hatching occurred near the ICM, we observed embryos hatched completely with the inducement of U-shaped hatching and the appearance of a scattered ICM (Fig 6C). From this, we can infer that the effect of “8”-shaped hatching that produces a scattered ICM continued even after the blastocysts hatched completely. A scattered ICM was previously reported to increase fetal loss in mice [2], whereas an enlarged ICM was reported to increase the implantation rate in the case of human embryos [11]. In terms of the underlying causes of these differences, the ICM cell number has not been considered in relation to the enlarged ICM in previous studies; the ICM cell number served as a key viability index in a mouse study [12]. Thus, given the development of TLM in recent years, it would be desirable to investigate clinical pregnancy prognoses by using specific TLM-based evaluations of the ICM.

In this study, we observed that in the “8”-shaped hatching that occurred near the ICM, which resulted in a scattered ICM, a gap was visible between the Oct3/4-positive cells (Fig 5C). Previous work has indicated that the ICM grade or cell-adhesion looseness is related to MZT development [13], and in mouse, a delay in inter-cell adhesion has been shown to increase ICM division [14]. Because the scattered ICM arising from “8”-shaped hatching is considered to represent a state of cell-adhesion looseness, the occurrence of “8”-shaped hatching near the ICM could potentially be the factor that elicits MZT.

In mouse, a recent study showed that “8”-shaped hatching is more common *in vitro* than *in vivo* [5]. Moreover, in an *in vitro* culturing environment, hardening of the ZP was observed [15]. This could represent one cause of the occurrence of “8”-shaped hatching. Moreover, the ZP opening diameter at the hatching area appears to be related to the hatching morphology: previous work conducted on mouse embryos indicated that the small holes created in ICSI, assisted hatching, and other ZP operations formed “8”-shaped hatching and suggested the possibility that MZT would increase [16]. Furthermore, a human study indicated that when performing assisted hatching, if a small-diameter ZP hole is formed, the hatching time is extended or trapping occurs [4]. Similarly, as compared with U-shaped hatching, “8”-shaped hatching was reported to exhibit a lower hatch-completion rate during the observation period [3]. These findings support the view that when performing assisted hatching, creation of a large-diameter hole would be suitable for preventing “8”-shaped hatching.

Our results also suggest that “8”-shaped hatching can occur readily from near the ICM (Fig 1B(a-d)). Besides expansion and collapse of the blastocyst, enzymes produced by the TE [17], superoxide anion radicals [18], and the activity of actin filaments [19] have been reported to affect the hatching start site of mouse embryos. Why the difference in the hatching position appeared in our study is unclear, but this result is nevertheless highly intriguing.

The strong bonding of the TE by tight junctions, adherens junctions, and desmosomes helps maintain the embryo morphology [20–22]. Na^+^/K^+^-ATPase and aquaporin transport water molecules into the embryo, which leads to embryo expansion [23, 24]. This process of embryo expansion has been linked to hatching and implantation. TE rupture has been reported to cause the liquid filling the blastocoel to leak outside the embryo, thereby leading to the occurrence of blastocyst collapse [25]. Collapse was reported to be related to low hatching rates in mouse [26] or low implantation rates in human [7]. Conversely, the formation of a slit in the ZP due to collapse was considered to represent a potential opportunity for hatching [3, 26]. Moreover, blastocyst collapse has been reported to potentially produce MZT due to ICM ectopic adhesion and subsequent proliferation [9, 27].

As mentioned earlier, the collapse that occurs inside the ZP has been investigated previously, but no reports have been published on a collapse outside the ZP, such as the one that occurs in “8”-shaped hatching. Here, when we used TLM to examine the outside blastocyst, we observed the same type of collapse behavior as in the case of the inside blastocyst (S1 Movie). This behavior is also confirmed by previously obtained video recordings of human embryos [3]. We found that outside collapse in “8”-shaped hatching was considerably more common than inside collapse (Table 1). Nevertheless, between the inside and outside blastocysts, we detected no marked difference in the expression of the TE-structure proteins Cdx2, Na^+^/K^+^-ATPase, and ZO2 (Figs 4 and 9). This supports the possibility that outside collapse is very common because water molecules incorporated into the inside blastocyst flow out to the outside blastocyst. This appears to be related to the resistance of the ZP; because the outside blastocyst does not face any resistance to expansion by the ZP, it can expand readily. Moreover, the outside blastocyst could potentially exhibit repeated expansion and collapse because of the inflow of a considerable amount of water.

In this study, we detected a non-significant ICM spread effect due to outside collapse. However, when outside collapse was observed, Oct3/4-positive cells were found to be attached to the TE opposite the hatching site (Fig 8). Furthermore, outside collapse occurred more frequently than inside collapse, and it might thus facilitate ICM separation when the ICM was present in the outside blastocyst. Therefore, outside collapse appears to be a behavior that warrants further attention in the evaluation of ICM morphology.

The prognosis of the scattered ICM observed here is unknown. Similarly, the prognosis of the separated ICM caused by outside collapse is unknown, as is whether or not the separated ICM leads to MZT. To test these possibilities, pregnancy rates and birth prognoses following blastocyst transfer will have to be investigated. Furthermore, we believe that it is essential to study the relationship between “8”-shaped hatching and ICM formation in human embryos in the future.

Blastocyst transfer has been increasingly performed in recent years. To perform blastocyst transfer, the *in vitro* period is extended, and thus “8”-shaped-hatching blastocysts can also be expected to increase in the future. In the evaluation of the “8”-shaped-hatching blastocysts, hatching position and outside collapse could emerge as key indices for embryo selection.

## Supporting information

S1 Movie Time-lapse movie of an “8”-shaped-hatching blastocyst. (MP4)

S2 Movie Time-lapse movie of a “U”-shaped-hatching blastocyst. (MP4)

S3 Movie Immunostaining at 120 h post-insemination. Oct3/4 (red) and Cdx2 (green) were stained with specific antibodies, and nuclei were stained with Hoechst (blue).
(MP4)
